# Supplementary material for: A UMLS-based spell checker for natural language processing in vaccine safety
Source: BMC Med Inform Decis Mak. 2007 Feb 12;7:3. doi: 10.1186/1472-6947-7-3 (PMC1805499; doi:10.1186/1472-6947-7-3)
Supplement: Additional file 13 — RAP application directory. Sets up directory for RAP (RDF application for PHP) source code [file 1472-6947-7-3-S13.gz › rap/doc/phpdoc/classtrees_ontModel.html]

xml version="1.0" encoding="iso-8859-1"?


## Root class OntModel

- ResModel **(Different package)**
  - OntModel

## Root class OntResource

- ResResource **(Different package)**
  - OntResource
    - Individual
    - OntClass
    - OntProperty

## Root class OntVocabulary

- OntVocabulary
  - OWLVocabulary
  - RdfsVocabulary

Documentation generated on Thu, 7 Jul 2005 13:41:08 +0200 by phpDocumentor 1.3.0RC3
